# Nutritional value of several commercially important river fish species from the Czech Republic

**DOI:** 10.7717/peerj.5729

**Published:** 2018-10-12

**Authors:** Sarvenaz Khalili Tilami, Sabine Sampels, Tomáš Zajíc, Jakub Krejsa, Jan Másílko, Jan Mráz

**Affiliations:** 1 Institute of Aquaculture and Protection of Waters, University of South Bohemia in České Budějovice, Faculty of Fisheries and Protection of Waters, South Bohemian Research Centre of Aquaculture and Biodiversity of Hydrocenoses, České Budějovice, Czech Republic; 2 Department of Molecular Sciences, BioCenter, Swedish University of Agricultural Sciences, Uppsala, Sweden

**Keywords:** Eicosapentenoic acid, Docosahexaenoic acid, Nutritional value, Index of atherogenicity, Index of thrombogenicity

## Abstract

Proximate and fatty acid (FA) composition of seven freshwater fish species from the Czech Republic were examined. Moreover, the index of atherogenicity (IA) and the index of thrombogenicity (IT) were calculated from the obtained data. These two indices along with the total content of the essential n-3 FAs, eicosapentaenoic acid (EPA) and docosahexaenoic acid (DHA) as well as the ratio of n-6/n3 FAs, provide good indicators for the nutritional value of the fish. The species had been selected owing to the limited amount of information about their nutritional composition available. Furthermore, they are not typically subject to aquaculture, being almost exclusively obtained by angling. The protein content was relatively stable in all species (17.1 ± 1.55 to 19.2 ± 2.20 g/100 g). The content of carbohydrates ranged from 0.02 ± 0.1 to 0.99 ± 0.0 g/100 g and ash from 1.08 ± 0.20 to 2.54 ± 1.57 g/100 g. As expected, a high variability was observed in the fat content (0.74 ± 0.04 to 4.04 ± 0.81 g/100 g) and the FA composition, as well as the contents of EPA and DHA. IA and IT were close to the values stated for the Eskimo diet, indicating a high nutritional value with a positive effect for human health.

## Introduction

The consumption of fish as well as fish products has significantly increased during the last two decades (Food and Agriculture Organization (FAO), 2016). The popularity of fish is mainly due to the overall high quality and the positive effects on human health. The main health benefits of fish are attributed to their high content of n-3 long-chain polyunsaturated fatty acids (FAs) (n-3 LC-PUFA) (Kris-Etherton et al., 2002; Lund, 2013; Khalili Tilami & Sampels, 2018). The most important n-3 LC-PUFA are eicosapentaenoic acid (EPA) and docosahexaenoic acid (DHA), which are known to have positive effects on the cardiovascular system as well as the nervous system of children in prenatal development, and to prevent the metabolic syndrome or obesity (Williams, 2000; Calder & Yaqoob, 2009; Adamkova et al., 2011; Torris, Molin & Cvancarova Smastuen, 2016; Kanakri et al., 2017; Saini & Keum, 2018). More recently fish proteins, peptides and amino acids have also gained increased attention with similar properties to the n-3 FAs from fish (Khalili Tilami & Sampels, 2018).

Moreover, fish proteins are easily digestible and rich in all essential amino acids, particularly methionine, lysine, taurine, which are limited in other kinds of muscle food (Tacon & Metian, 2013; Khalili Tilami & Sampels, 2018). Khalili Tilami & Sampels (2018) provided a depth review of the nutritional value of fish, focusing on lipids and proteins in particular.

While the protein composition is generally very stable in fish, the FA composition is greatly influenced by the diet. The lipid composition of the diet is mirrored in the fillet lipid composition of reared fish following the trend “You are what you eat” (Chanmugam, Boudreau & Hwang, 1986; Sahena et al., 2009). In addition to the effect of feed composition, other factors including fish feeding habits, fish trophic level and ecosystem trophic status might influence the nutritional composition of fish in natural water bodies via changing the quality of feed sources (Ahlgren et al., 1996; Czesny et al., 2011; Gladyshev et al., 2018). Nutritional composition of various fish species might be influenced by variation in their morphology and physiology (Rust, 2002; Khitouni et al., 2014).

The differences in nutrient composition between wild and farmed fish of identical species have been reported many times (Nettleton & Exler, 1992; Ahlgren, Carlstein & Gustafsson, 1999; Orban et al., 2003; Kaushik et al., 2006; Hossain, 2011). The diets for fish in intensive aquaculture consist of complete feeding mixtures based on fish meal and fish oil to meet the fish requirements as well as reaching a nutritionally valuable high content of n-3 FA in the fillet. Nonetheless, due to the increased use in various sectors (aquaculture, pharmaceuticals, cosmetics...), n-3 LC-PUFA rich sources for aquaculture feeds are very limited and must be replaced by sustainable components. For the time being, these replacers are usually plant components, generally causing a decrease in the proportion of n-3 LC-PUFA in the fish. On the contrary, the diet of wild fish consists of natural feed, such as plankton, benthos as well as nekton in case of carnivorous species, which naturally contain the essential n-3 LC-PUFA. The primary producers of n-3 LC-PUFA in freshwater ecosystems are, the same as in the ocean, algae. These compounds are transferred into the fish throughout the feed chain. In addition, fish are able to biosynthesize n-3 LC-PUFA from their 18 carbon precursor (α-linolenic acid; ALA) to a certain degree. This ability is strongly expressed in freshwater non-carnivorous species, compared to marine carnivorous fish, which decreased this ability during evolution (Tocher, 2003; Zajic, Mraz & Pickova, 2016). Therefore, the consumption of freshwater species from natural habitats should be beneficial not only for human health, but also from sustainability and ecological viewpoints.

While annual fish supply around the world was 20 kg per capita in 2014 (Food and Agriculture Organization (FAO), 2016), landlocked countries, like those in Central Europe, have a much lower average consumption; for instance fish intake in the Czech Republic was around 5.5 kg per capita in 2008 (MZe, 2009). At the same time, a significant percentage of consumed fish is provided by anglers in these countries, thus consisting of wild fish. The consumed fish also include species that have unjustly gained less attention by experts for human nutrition. However, they have a relatively high importance for a certain part of the population in Central Europe.

This study aimed to complement the existing information about the nutritional composition and lipid indices of seven less promoted but very interesting freshwater fish species in order to extend an existing knowledge. For some of them no relevant data about proximate composition exist and only fragmentary results have been published regarding the fat content and composition. The list of investigated species includes European grayling (*Thymallus thymallus*), common nase (*Chondrostoma nasus*), brown trout (*Salmo trutta morpha fario*), common bream (*Abramis brama*), Prussian carp (*Carassius gibelio*), European perch (*Perca fluviatilis*) and European chub (*Squalius cephalus*).

## Materials and Methods

The fish (individuals of a consumerist size) for this study were obtained by anglers from their natural habitat (major river basin of the Dyje, Labe and Vltava rivers in the Czech Republic) during the vegetation season. Samples from each species were caught at different localities. After catching of each fish by traditional angling (angle with one hook attached to the fishing line), fish were separated based on their weight. Then, the individuals with the marketable-size were selected. Immediately after capture, the selected fish were killed by a blow to the head, weighted (Table 1) and transported on ice (0 °C) to the processing facilities of the Institute of Aquaculture and Protection of Waters, Faculty of Fisheries and Protection of Waters, University of South Bohemia, Ceske Budejovice, Czech Republic. The temperature was monitored during the transport. Fish were filleted and processed as skin-on and scale-less. Fillet with skin were used in order to include all the flesh and FA deposits which contain n-3 LC-PUFA. Then the whole remaining fillet was homogenized in a table blender so that the taken sample was sufficiently representative, while containing all the edible parts.

**Table 1:** List of seven analyzed freshwater fish species from major river basin of Dyje, Labe and Vltava river, the Czech Republic, with weight (average ± standard deviation) and captured fish number (*N*).

Common name	Latin name	Average weight (g)	*N*
Freshwater bream	*Abramis brama*	761 ± 158	16
European perch	*Perca fluviatilis*	142 ± 29	10
Prussian carp	*Carassius gibelio*	483 ± 96	13
Common nase	*Chondrostoma nasus*	510 ± 115	10
Brown trout	*Salmo trutta morpha fario*	140 ± 48	12
Grayling	*Thymallus thymallus*	315 ± 44	13
Chub	*Squalius cephalus*	243 ± 35	11

**Note:**

*N*-captured fish number.

### Proximate composition

The chemical composition of fish samples was analyzed following standardized AOAC (Association of Official Analytical Chemists (AOAC), 2000) methods. For dry matter analysis, 12 individuals of common bream, seven European perch, eight Prussian carp, seven common nase, 12 brown trout, nine European grayling, and five European chub were used. To determine dry matter, five g of homogenized sample was mixed with some sea sand in a pre-dried porcelain dish and then dried in the oven at a temperature of 105 °C to the constant weight. A total of 12 individuals of common bream, 10 European perch, nine Prussian carp, nine common nase, 12 brown trout, 13 European grayling, and 11 European chub were taken for ash analysis. Ash was analyzed by incinerating five g homogenized muscle at 550 °C in a muffle furnace for 12 h. Carbohydrates were calculated using the following formula:

$${\rm{Carbohydrates }}\ \left( {\rm{\% }} \right) = {\rm{100}}-\left( {{\rm{moisture}} + {\rm{lipids}} + {\rm{proteins}} + {\rm{ash}}} \right)$$

For protein analysis, 12 individuals of common bream, ten European perch, nine Prussian carp, nine common nase, nine brown trout, 12 European grayling, and 10 European chub were used. Total nitrogen was analyzed in a certified laboratory (ALS Czech Republic, Prague) by Dumas combustion, the protein content being subsequently calculated using 6.25 as a conversion factor. The energy value was calculated assuming conversion factors of 23.6, 39.5, and 17.2 kJ/100 g for proteins, lipids, and carbohydrates, respectively (NRC, 1993).

### Fat content and fatty acid composition

A total of 15 individuals of common bream, 10 European perch, 13 Prussian carp, nine common nase, 12 brown trout, 10 European grayling, and 10 European chub were used for analysis. One g of the homogenized fillet was taken for analysis. Lipids were extracted in HIP (hexane-isopropanol 3:2 *v:v*) following the method of Hara & Radin (1978) with modifications described by Mraz & Pickova (2009) and the fat content was determined gravimetrically. Subsequently fatty acid methyl esters (FAME) were prepared according to Appelqvist (1968) with NaOH in dry methanol and boron trifluoride–methanol complex (BF_3_). Obtained FAMEs were analyzed using the gas chromatograph Trace Ultra (ThermoScientific, Waltham, MA, USA) equipped with a flame ionization detector and capillary column BPX 70 (AGE, Austin, TX, USA) with 50 m length × 0.22 mm i.d. × 0.25 μm film thickness. FA were identified by comparing to the standard mixture GLC-68D (Nu-Check Prep, Elysian, MN, USA) and other individual standards. For calculations of the absolute amount of individual FA, an internal standard (21:0) (Nu-check Prep, Elysian, MN, USA) was used.

### Lipid health indices

The obtained data were used to calculate both the index of atherogenicity (IA) and the index of thrombogenicity (IT) according to Ulbricht & Southgate (1991). The IA refers to the ratio between the main saturated FA (SFA) and the sum of monounsaturated FA (MUFA), and polyunsaturated FA (PUFA). The result of this index is a number indicating the risk of formation i.e., atherosclerosis. The higher the IA is, the higher risk it constitutes. The IT is defined as the ratio between pro-thrombogenic (myristic, palmitic, and stearic) and anti-thrombogenic (MUFA, n-6 PUFA and n-3 PUFA) FA. An increasing IT indicates a risk of developing a blood clot (Garaffo et al., 2011; Ulbricht & Southgate, 1991). The following equations were applied:

$$\matrix{{{\rm{IA}} = \left( {12{\rm{:}}0 + 4 \times 14{\rm{:}}0 + 16{\rm{:}}0} \right)/\left[ {{\rm{\Sigma MUFA}} + {\rm{\Sigma PUFA}}} \right]} \hfill \cr \matrix{{\rm{IT}} = \left[ {14{\rm{:}}0 + 16{\rm{:}}0 + 18{\rm{:}}0} \right]/[\left( {0.5 \times {\rm{MUFA}}} \right) + \left( {0.5 \times {\rm{n}} - 6} \right) + \left( {3 \times {\rm{n}} - 3} \right) \hfill \cr \quad \;\;\; + \left( {{\rm{n}} - 3/{\rm{n}} - 6} \right)] \hfill \cr} \hfill \cr } $$

### Statistical analysis

Statistical evaluation was performed using one-way analysis of variance (ANOVA) with subsequent post hoc comparisons using Tukey’s honest significant difference test to determine the effects of different localities on the changes of FAs, lipids, proteins, dry matter, and carbohydrates within species. Probability values of *p* ≤ 0.05 were considered as significant. These statistical analyses were performed using the STATISTICA software (Version 13; StatSoft, Inc., Tulsa, OK, USA) for MS Windows. The relation between FAs and lipid content of each species were evaluated using linear regression. Kruskal Wallis one-way ANOVA was performed in order to determine differences in FAs, dry matter, proteins, lipids, and ash content among fish species. In case of significant differences Dunn post hoc test were performed. These analyses were done with rcompanion (Magniafico, 2018) and FSA (Ogle, 2018) packages in R version 3.4.4 (R Development Core Team, 2018).

## Results

The present study analyzed the fillet composition of seven wild freshwater fish species. The fish species presented in this study are normally solely captured from open waters with exception of European perch (Mairesse et al., 2006) and to a limited extent brown trout (Arzel et al., 1994). The obtained samples originated exclusively from the natural conditions of the species studied, from the Dyje, Labe, and Vltava river basins. The purpose was to only take the fish that had reached the consumable size (Table 1), as the nutrient composition with lipids in particular can vary with the growth of the fish (Mraz & Pickova, 2011). It has to be considered that beside growth and the already earlier mentioned feed composition many other factors can influence nutrient composition in fish. For example, ecological factors including the trophic status of the water body (eutrophic ecosystem enriched with phytoplankton as the main producers of feed chain versus oligotrophic ecosystem) (biotic factor) (Ahlgren et al., 1996; Czesny et al., 2011; Vasconi et al., 2015, Gladyshev et al., 2018), temperature (Arts et al., 2012) as well as lightning conditions (abiotic factors) (Boujard & Leatherland, 1992) were reported to have influence on FA and lipid composition of the fish by changing the quality of their feed. Other factors like fish feeding habits, their preference for eating, presence and threat of predation (Daan, 1981) which can change the fish preferred time of feeding in spite of their fixed feeding rhythms as diurnal or nocturnal feeders, are also important. The role of phylogenetic factor is more discussed for carnivorous species (Gladyshev et al., 2018). However, in the present study the aim was to investigate the natural composition and possible diversity in order to be able to give better information about nutritional composition to the consumers. Therefore, these factors were not in the focus of the work.

### Proximate composition

The proximate composition of the analyzed fish is listed in Table 2. Fat content varied from 0.74% in European perch to 4.04% in common nase. All studied species showed a similar protein content (17.1 ± 1.55 to 19.2 ± 2.20 g/100 g fillet). The carbohydrate content was varying from 0.02 g/100 g (European perch) to 0.9 g/100 g (common bream). Like proteins and carbohydrates, the ash content in the fillet with skin was comparable among all the analyzed species (1.08 ± 0.20 to 2.54 ± 1.57 g/100 g).

**Table 2:** Proximate composition of seven freshwater fish species from major river basin of Dyje, Labe and Vltava river, the Czech Republic.

	Dry matter	Protein	Lipids	Ash	Carbohydrate	Energy value	Energy value
	g/100 g	g/100 g	g/100 g	g/100 g	g/100 g	kJ/100 g	kcal/100 g
Common bream	22.5 ± 1.85^a^	18.0 ± 1.24^ab^	2.17 ± 0.19^a^	1.35 ± 0.18^bc^	0.99 ± 0.0^a^	528 ± 18^ab^	126 ± 4^ab^
European perch	20.9 ± 2.67^c^	17.6 ± 1.85^bc^	0.74 ± 0.04^c^	2.54 ± 1.57^ab^	0.02 ± 0.1^e^	500 ± 31^b^	114 ± 2^b^
Prussian carp	20.8 ± 1.41^ac^	17.1 ± 1.55^c^	1.94 ± 1.13^a^	1.08 ± 0.20^c^	0.68 ± 0.0^ab^	518 ± 4^ab^	124 ± 1^ab^
Common nase	23.4 ± 1.47^bd^	17.6 ± 0.98^bc^	4.04 ± 0.81^b^	1.25 ± 0.08^bc^	0.51 ± 0.1^bc^	604 ± 58^ac^	144 ± 14^ac^
Brown trout	24.3 ± 1.50^b^	19.2 ± 1.50^a^	3.32 ± 0.1^ab^	1.56 ± 0.20^abc^	0.30 ± 0.1^df^	619 ± 56^c^	148 ± 13^c^
European grayling	21.6 ± 1.94^ad^	17.4 ± 0.52^bc^	2.77 ± 0.92^ab^	2.35 ± 1.05^a^	0.18 ± 0.1^ef^	536 ± 33^ac^	128 ± 8^ac^
European chub	24.9 ± 0.2^b^	19.2 ± 2.20^ab^	3.49 ± 0.53^b^	1.86 ± 0.51^ab^	0.37 ± 0.0^cd^	611 ± 76^ac^	146 ± 18^ac^

**Notes:**

Different letters indicated significant differences (*p* ≤ 0.05) for the respective parameter among different species.

Data are mean ± standard deviation.

### Fatty acid composition

**Figure 1:**
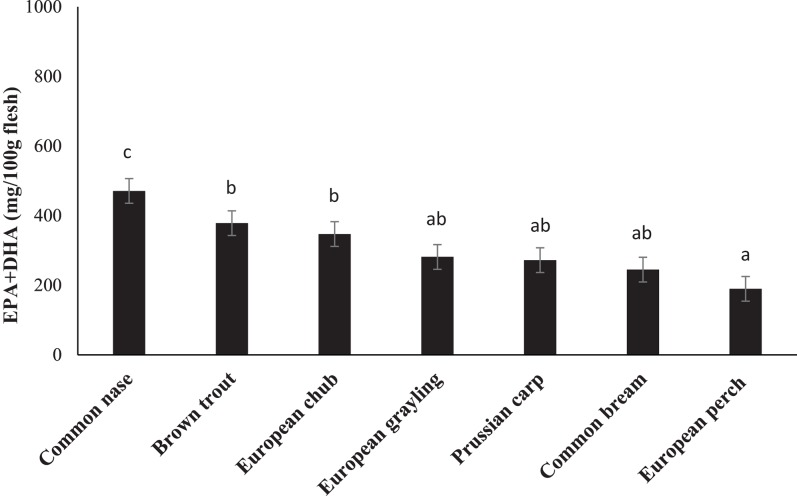
The content (mg/100 g flesh) of eicosapentaenoic (EPA; 20:5n-3) and docosahexaenoic (DHA; 22:6n-3) acids in the fillet of seven freshwater fish species from the major river basin of Dyje, Labe and Vltava river, the Czech Republic. Data are the mean ± standard deviation. Different letters indicate significant differences among species (*p* ≤ 0.05).

Fatty acid composition of the chosen species is presented in Table 3. FA composition varied between species. The nutritional valuable n-3 FA, EPA, and DHA showed values between 2.03% in brown trout and 8.15% in common bream for EPA and 7.33 in common nase to 27.60% in European perch for DHA. Total content of EPA plus DHA was calculated to range from 190 mg/100 g in European perch to 471 mg/100 g in common nase (Fig. 1).

**Table 3:** Fatty acid composition (% of total identified), atherogenicity and thrombogenicity indices of seven freshwater fish species caught from the major river basin of Dyje, Labe and Vltava river, the Czech Republic.

	Common bream	European perch	Prussian carp	Common nase	Brown trout	European grayling	European chub
14:0	2.02 ± 0.8^a^	0.99 ± 0.22^b^	2.15 ± 0.46^a^	2.69 ± 0.88^a^	1.95 ± 0.58^a^	1.94 ± 0.57^a^	1.93 ± 0.29^a^
14:1	0.62 ± 0.45^a^	0.32 ± 0.35^c^	1.26 ± 2.19^ab^	0.22 ± 0.09^c^	0.32 ± 0.15^abc^	0.06 ± 0.08^bc^	0.58 ± 0.15^a^
16:0	14.3 ± 6.82^ab^	22.38 ± 3.35^c^	17.31 ± 2.83^ab^	16.19 ± 2.82^bd^	18.57 ± 4.20^ac^	12.89 ± 2.79^d^	17.09 ± 1.11^ab^
16:1	9.78 ± 5.33^bc^	3.85 ± 1.33^a^	7.81 ± 2.10^ac^	15.00 ± 4.73^b^	7.92 ± 2.72^ac^	3.57 ± 1.31^a^	9.99 ± 2.05^bc^
18:0	5.98 ± 2.13^a^	4.47 ± 2.35^ac^	2.83 ± 2.54^bc^	3.09 ± 0.36^bc^	4.67 ± 1.07^b^	2.87 ± 0.58^ab^	3.26 ± 0.42^bc^
18:1n-9	17.9 ± 9.19^a^	7.93 ± 2.93^b^	7.33 ± 5.67^b^	17.12 ± 1.95^a^	22.94 ± 15.9^a^	27.30 ± 9.39^c^	21.23 ± 2.47^ac^
18:1n-7	6.53 ± 1.65^a^	3.85 ± 0.33^bc^	4.99 ± 0.49^ab^	4.81 ± 0.07^ab^	3.97 ± 1.27^abc^	2.83 ± 0.30^c^	6.01 ± 0.13^a^
18:2n-6	7.63 ± 2.51^a^	3.22 ± 0.39^b^	7.12 ± 3.00^a^	5.14 ± 1.45^a^	5.91 ± 2.47^a^	16.29 ± 2.87^c^	8.23 ± 4.16^a^
18:3n-3	3.61 ± 1.61^ab^	1.94 ± 1.12^c^	5.35 ± 2.63^a^	2.29 ± 1.14^bc^	6.75 ± 4.37^ab^	2.54 ± 0.70^bc^	4.99 ± 0.50^a^
20:0	0.46 ± 0.22^a^	0.15 ± 0.06^c^	0.28 ± 0.1^abc^	0.20 ± 0.0^abc^	0.38 ± 0.1^abc^	0.14 ± 0.1^bc^	0.35 ± 0.1^ab^
20:1n-9	0.70 ± 0.28^ab^	0.81 ± 0.91^b^	1.41 ± 0.32^ac^	2.09 ± 2.85^cd^	0.80 ± 0.56^ac^	2.63 ± 0.61^d^	0.92 ± 0.11^c^
20:2n-6	1.16 ± 0.37^ab^	0.43 ± 0.37^c^	3.79 ± 2.43^a^	0.40 ± 0.10^cd^	0.68 ± 0.76^cd^	1.12 ± 1.69^bd^	1.04 ± 0.93^bcd^
20:4n-6	6.12 ± 3.65^a^	7.89 ± 2.97^a^	2.41 ± 3.83^bd^	2.41 ± 3.83^bd^	2.73 ± 1.38^bc^	1.10 ± 0.57^d^	3.87 ± 0.63^ac^
20:3n-3	0.62 ± 0.22^ab^	0.35 ± 0.19^d^	3.31 ± 2.10^c^	0.40 ± 0.20^ad^	0.61 ± 0.22^ab^	0.19 ± 0.04^d^	0.82 ± 0.17^bc^
22:0	0.09 ± 0.09^a^	0.03 ± 0.04^c^	0.08 ± 0.04^abc^	0.07 ± 0.04^abc^	0.20 ± 0.06^abc^	0.00 ± 0.00^bc^	0.01 ± 0.02^ab^
22:1	0.56 ± 0.98^ab^	1.04 ± 1.29^b^	0.75 ± 0.54^a^	0.60 ± 0.45^ab^	0.88 ± 0.59^a^	0.35 ± 0.09^ab^	0.81 ± 1.20^ab^
20:5n-3	8.15 ± 11.6^a^	4.45 ± 1.75^a^	3.85 ± 1.79^ac^	6.82 ± 5.02^a^	2.03 ± 0.92^b^	2.32 ± 1.54^bc^	3.39 ± 1.48^abc^
24:1	0.52 ± 0.66^a^	1.04 ± 1.29^a^	0.87 ± 0.78^a^	2.15 ± 1.68^a^	1.25 ± 0.91^a^	0.40 ± 0.32^a^	0.49 ± 0.57^a^
22:5n-3	2.85 ± 1.26^a^	2.48 ± 0.26^a^	2.72 ± 0.68^a^	3.02 ± 0.68^a^	1.60 ± 0.43^b^	1.08 ± 0.36^b^	1.89 ± 0.31^b^
22:6n-3	7.68 ± 4.43^a^	27.60 ± 3.61^d^	13.19 ± 5.00^b^	7.33 ± 2.37^ac^	12.83 ± 8.76^abc^	12.95 ± 7.33^bc^	10.48 ± 4.23^a^
24:0	6.31 ± 2.23^a^	1.01 ± 0.33^abc^	0.70 ± 0.25^bc^	0.24 ± 0.04^b^	0.53 ± 0.40^b^	2.06 ± 0.41^ac^	1.53 ± 1.70^bc^
SFA	25.7 ± 5.51^ab^	28.30 ± 5.05^b^	22.88 ± 4.41^ac^	22.24 ± 3.65^ac^	25.72 ± 5.18^ab^	17.75 ± 4.34^c^	23.23 ± 1.26^ab^
MUFA	37.9 ± 10.6^a^	21.77 ± 7.01^c^	35.59 ± 11.11^ab^	42.17 ± 5.53^b^	38.71 ± 13.35^ab^	45.75 ± 8.63^b^	40.62 ± 3.03^ab^
PUFA	37.8 ± 11.4^a^	48.34 ± 4.31^c^	41.33 ± 6.10^ac^	27.80 ± 5.91^b^	33.15 ± 8.72^ab^	37.80 ± 7.07^a^	34.71 ± 2.80^ab^
n-3 PUFA	22.9 ± 11.3^a^	36.80 ± 3.80^c^	28.42 ± 4.55^bc^	19.86 ± 5.13^a^	23.83 ± 8.79^ab^	19.07 ± 8.40^a^	21.57 ± 3.09^ab^
n-6 PUFA	14.9 ± 3.93^ab^	11.54 ± 2.68^c^	12.91 ± 2.89^ac^	7.94 ± 4.93^d^	9.32 ± 3.74^cd^	18.73 ± 3.09^b^	13.14 ± 3.98^ac^
n-3 HUFA	19.3 ± 11.8^a^	34.86 ± 3.64^c^	23.07 ± 6.27^bc^	17.57 ± 4.49^a^	17.08 ± 8.72^ab^	16.53 ± 8.95^a^	7.21 ± 6. 88^ab^
n-3/n-6	1.74 ± 1.61^ab^	3.40 ± 1.10^ce^	2.29 ± 0.54^ade^	3.19 ± 1.84^cde^	2.95 ± 1.39^c^	1.10 ± 0.76^b^	1.88 ± 0.81^ad^
IA	0.30 ± 0.11^a^	0.38 ± 0.06^a^	0.35 ± 0.07^a^	0.39 ± 0.09^a^	0.37 ± 0.10^a^	0.07 ± 0.30^b^	0.35 ± 0.02^ab^
IT	0.25 ± 0.10^ab^	0.22 ± 0.04^ab^	0.20 ± 0.05^ab^	0.25 ± 0.02^a^	0.26 ± 0.07^a^	0.06 ± 0.22^b^	0.25 ± 0.03^ab^

**Notes:**

Data are presented as mean ± standard deviation.

Different letters indicate significant differences (*p* ≤ 0.05) for the respective FA among different species.

IA, index of atherogenicity; IT, index of thrombogenicity; MUFA, monounsaturated fatty acids; PUFA, polyunsaturated fatty acids; SFA, saturated fatty acids.

### Lipid health indices

One of the sub-objectives of this study was to determine the lipid health indices (IA and IT) of the analyzed species. In this study, the IA reached a maximum of 0.39 (common nase) and the highest IT (0.26) was calculated for brown trout (Table 3).

## Discussion

Protein content results varied at the predictable levels and corresponded to the indicated values for fish flesh (Lazos, Aggelousis & Alexakis, 1989; Puwastien et al., 1999; Tuomisto & Froyland, 2008; Gjedrem, Robinson & Rye, 2012; Zotos & Vouzanidou, 2012). Carbohydrate content in fish is usually lower than 0.5 g/100 g flesh (Gjedrem, Robinson & Rye, 2012). Ash content varied from 1.08 to 2.54, this might be due to the variation in feed intake, species, physiology, and sex (Khitouni et al., 2014). Similar variations have been observed in the results of previous studies (Puwastien et al., 1999; Zotos & Vouzanidou, 2012; Zivkovic et al., 2013). As protein and carbohydrate contents are known to be very stable in fish, these results had been expected (Morris, 2001; Shearer, 2001). Significant differences occurred only earlier when the whole-body composition (with bones, fins, and scales) of fish was analyzed (Van Pelt et al., 1997).

Common bream is a lean fish with approximately one g of lipids per 100 g fillet (Lazos, Aggelousis & Alexakis, 1989; Aggelousis & Lazos, 1991). In our study we found fat contents up to 2.17 ± 0.19, which is rather comparable with the North European populations of this species with 1.8 g/100 g (Puustinen, Punnonen & Uotila, 1985). Even higher fat levels (3.63–5.51 g/100 g) are published by Zmijewski et al. (2006) and Zivkovic et al. (2013). This variability in fat content is consequently accompanied by differences in FA composition, as the relative content of n-3 LC-PUFA generally decreases with an increasing fat content, as storage fat is built by triacylglycerols (TAG), which are usually higher in SFA and MUFA (Henderson & Tocher, 1987). Common bream showed to have a nutritionally very favorable FA composition, with high proportions of n-3 LC-PUFA (Table 3). This most probably reflected the composition of the natural diet, as diet FA composition was shown earlier to be the most important factor influencing the fish muscle composition (Robin et al., 2003; Pickova, Sampels & Berntsen, 2010). Common bream as a benthos- and plankton feeders species with the nocturnal feeding habits has a vast feeding spectrum which can feed on detritus, mollusks and macrophytes (Adamek & Marsalek, 2012; Zapletal et al., 2012; Golovanova et al., 2014). They belong to the higher trophic levels therefore, digestion for them is not as easy as for herbivorous species. This might influence the metabolic apparatuses and fish FA composition (Rodrigues et al., 2017). Considering the differences between the intestine length and morphology of various fish species and their consequent effects on the intestine absorptive surface and digestibility of the feed (Rust, 2002), nutritional composition of fish species might be influenced.

**Figure 2:**
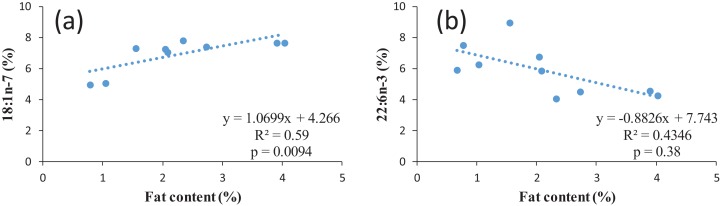
Examples of regression between lipid content and two FAs in common bream: (A) 18:1n-7; (B) 22:n-3.

According to the fillet fat content, the proportion of n-3 LC-PUFA in similar studies varies widely from 4.7 up to 31.8%. The n-3/n-6 ratio could be close to one (Zivkovic et al., 2013), around 1.7 (present study) or up to 2.9 (Aggelousis & Lazos, 1991) also indicating the effect of feed composition as the natural feed composition most probably varies in different water bodies. However, all values are within the recommended values of a n-6/n-3 ratio of 1–4 (Simopoulos, 2008). When discussing the nutritional value of fish for human, it must be observed that normally the ratio between n-6 and n-3 FAs in food items and in nutrition is expressed as n-6/n-3, while in fish the ratio between is often expressed as n-3/n-6, since the opposite ratio would lead to very low values below 0. For example, the n-3/n-6 ratio of 1.7 for common bream in the present study corresponds to n-6/n-3 ratio of 0.65. The regression between the FAs and lipid content of common bream was investigated. In terms of individual FA, in case of MUFA with an increase in the percentage of the fat content, the percentage of some MUFAs including 14:1 (*p* = 0.02, *R*_2_ = 0.49); 16:1 (*p* = 0.02, *R*_2_ = 0.49); 18:1n-7 (*p* = 0.009, *R*_2_ = 0.59) significantly increased (positive correlation) (Fig. 2A as an example for one of the MUFAs) whereas in 22:6n-3 (*p* = 0.03, *R*_2_ = 0.43), with an increase in the percentage of the fat content, a significant decrease in this PUFAs percentage was observed (inverse correlation) (Fig. 2B). This confirms the earlier mentioned fact that a higher fat content also corresponds to a lower LC-PUFA percentage. In general, the fat is stored in TAG, resulting in an increase proportion of this lipid fraction in fatty fish, while in general LC-PUFA are stored in the phospholipids, which are mainly the constituents of biological membranes (Sargent et al., 1999). As a higher proportion of TAG automatically results in a (relative) lower proportion of phospholipids, this will also lead to a relatively lower proportion of LC-PUFA (Henderson & Tocher, 1987).

Similarly as for common bream, lower fat contents compared to the present work were stated also for European chub by Lazos, Aggelousis & Alexakis (1989) and Aggelousis & Lazos (1991) (average of 1.5 g/100 g compared to 3.49 ± 0.5 g/100 g in the present study). On the other hand, Donmez (2009) found similar values to 3.5 ± 0.4 g/100 g. Based on the available data, the n-3/n-6 ratio in European chub showed to vary from 1.7 (Aggelousis & Lazos, 1991) through 1.9 (present study) up to 2.7 (Donmez, 2009). The differences are most likely caused by feed available in the habitat, as the fish, being an omnivorous species with their shallow water preferences, can eat everything from fallen fruit to small fish (Piria et al., 2005), which subsequently influences the fillet fat content and FA composition.

In addition, the effect of different localities on the changes in the proximate composition in European chub was tested. No significant changes in the parameters were observed. The regression between the lipid content and several FAs including 14:0 (*p* = 0.002, *R*_2_ = 0.69); 16:0 (*p* = 0.002, *R*_2_ = 0.70); 18:1n-9 (*p* = 0.020, *R*_2_ = 0.50); 18:2n-6 (*p* = 0.029, *R*_2_ = 0.46); 18:3n-3 (*p* = 0.01, *R*_2_ = 0.57); 20:1n-9 (*p* = 0.005, *R*_2_ = 0.63); 20:5n-3 (*p* = 0,09, *R*_2_ = 0.30) showed positive correlation, whereas in 14:1 (*p* = 0.0005, *R*_2_ = 0.79); 20:3n-3 (*p* = 0.07, *R*_2_ = 0.33); 24:1 (*p* = 0.003, *R*_2_ = 0.67); 22:5n-3 (*p* = 0.07, *R*_2_ = 0.34); 22:6n-3 (*p* = 0.00009, *R*_2_ = 0.86) negative correlation was observed, again confirming the correlation between a higher fat content and increased SFA content as mentioned before.

The feed of common nase, which is herbivorous benthopelagic species (Junger, Kotrschal & Goldschmid, 1988; Riede, 2004), may consist of indigestible material in a high portion which requires longer gut (Hofer, 1982). Previously, very low amounts of lipids (∼1 g/100 g) were found in the fillet of nase (Lazos, Aggelousis & Alexakis, 1989). A slightly higher content (1.3 ± 0.46 and 2.7 ± 0.4 g/100 g) was shown by Aggelousis & Lazos (1991) and Lazos (1997), respectively. On the other hand, our result showed much higher values with an average fat content of 4.04 ± 0.81 g/100 g. The differences could be attributed to the fact that the fish in the studies reporting a lower fillet fat content originated from Southern Europe with a higher average year-round water temperature. Subsequently, the fish do not need to store as much lipid as in the areas with a cold winter. Other noticeable findings for this species in the present study were high proportions of EPA and DHA (6.82 ± 5.02 and 7.33 ± 2.32%, respectively), which is, given the relatively low fat content, comparable with some marine species (Usydus et al., 2011). This is probably due to an exceptionally good nutritional environment of the investigated fish in combination with the fact that freshwater fish are able to convert 18 carbon precursors into their longer LC-PUFA derivatives (Tocher, 2003). Furthermore, Aggelousis & Lazos (1991) stated quite high values of these important FA (6% EPA and 9% DHA) in nase together with a very low-fat content as mentioned above. The effect of different localities on the proximate composition in nase showed significant differences in the ash content and moisture, whereas no significant changes were observed in the other parameters; this might be related to the feed and environment. The PUFA percentage tended to decrease significantly when total lipid content increased, such as in 20:4n-6 (*p* = 0.003, *R*_2_ = 0.68); 22:5n-3 (*p* = 0.013, *R*_2_ = 0.51); 22:6n-3 (*p* = 0.01, *R*_2_ = 0.53) inverse correlation were observed. Similar conclusions were obtained by Belling et al. (1997) and Zhang et al. (2014).

The only representative of a species which is captured as well as farmed in our study, is European perch. Our results confirm that wild European perch contains a minimum (0.3–1.5%) of fat in the fillet (Puustinen, Punnonen & Uotila, 1985; Mairesse et al., 2006; Orban et al., 2007), in our case (0.74 ± 0.04 g/100 g). Subsequently, due to the low-fat content, the relative percentage of n-3 LC-PUFA rises, which is confirmed by the highest percentage of these FA (36.80 ± 3.80%) of all species analyzed in this study. Olsson et al. (2007) found that the length of the gastrointestinal tract of European perch has an adaptive plasticity based on the different feed type they consume. This means that any changes in their diet leads to an individual specialization in the morphology of their digestive organs, particularly alteration in the relative length of the gastrointestinal tract can take place (Olsson et al., 2007). This is relevant as the natural habitat of the European perch (the littoral and pelagic habitats) has a high variation in the feed sources and in consequence results in differences in gastrointestinal tract length. The size of the digestive organs is connected with a more efficient use of the feed source (Sibly, 1981; Magnan & Stevens, 1993; Olsson et al., 2007).

European perch as an ichthyofagous/optional benthofagous species (Golovanova et al., 2014) is very popular among anglers in Central Europe and its high and appreciated flesh quality resulted in the beginning of farmed perch production. However, fish kept in recirculation systems usually have a higher fillet fat content, as the feeding intensity and the fat content of the feed can be higher than under natural conditions (Xu et al., 2001). Significant changes were observed in all parameters except dry matter, due to the effect of different localities indicating a high variation of feed composition and availability at the different localities. It could be also due to the sensitivity of European perch to the feed effects in general, since lipid is a major concern in European perch, which is greatly influenced by n-3 and n-6 FAs in the diet (Xu & Kestemont, 2002). Some FAs, including 14:0 (*p* = 0.09, *R*_2_ = 0.20); 16:1 (*p* = 0.0006, *R*_2_ = 0.63); 18:2n-6 (*p* = 0.042, *R*_2_ = 0.29) showed positive correlation and in case of 20:1n-9 (*p* = 0.09, *R*_2_ = 0.22) negative correlation to the fat content with significant changes was observed consequently due to the changes in fat content as a result of the effect of different localities.

European grayling is among the rarely consumed species, with an importance primarily in sport fishing. Additionally, its population is currently threatened in the region of Central Europe (Turek et al., 2014). Hence, European grayling is little known from a nutritional point of view. The fillet of European grayling showed to have a relatively low-fat content in line with earlier 2.3–2.6 g/100 g (Renaville et al., 2013); 2.77 ± 0.92 g/100 g in this study. According to our findings, there is a high proportion of n-3 PUFA (19.07 ± 8.40%) including EPA and DHA with relatively high variability, most probably again due to the respective available diet. Interestingly, the only similar study focused on the nutritional composition of wild and farmed European grayling brought substantially different results of FA composition compared to our results. While we found n-3/n-6 ratio of 1.10 ± 0.76, Ahlgren, Carlstein & Gustafsson (1999) described a ratio at 4–6 for wild and even 7–13 for farmed fish. The main difference was the high content of n-6 linoleic acid (16.29 ± 2.87%) in the fillet of European grayling from Central Europe. Comparable values were only presented in studies on Arctic grayling (*Thymallus arcticus*) published by Sushchik et al. (2006) and Gladyshev et al. (2012). Significant changes were observed for all parameters except protein content in connection with the changes in localities. Some FAs, including 16:0 (*p* = 0.059, *R*_2_ = 0.26); 18:0 (*p* = 0.01, *R*_2_ = 0.4); 20:4n-6 (*p* = 0.001, *R*_2_ = 0.56); 22:5n-3 (*p* = 0.0006, *R*_2_ = 0.63); 22:6n-3 (*p* = 0.0008, *R*_2_ = 0.62) showed an inverse correlation, to total fat content whereas a positive correlation was observed in FAs, including 16:1 (*p* = 0.06, *R*_2_ = 0.25); 18:1n-9 (*p* = 0.004, *R*_2_ = 0.5); 18:3n-3 (*p* = 0.006, *R*_2_ = 0.47). ALA is preferably stored in neutral lipid fraction, which is mainly consisting of TAG, hence it can increase with an increasing fat content (Enser et al., 2000).

In Central Europe, Prussian carp is an invasive carp-like species. We found a lower fat content (1.94 ± 1.13 g/100 g flesh) compared to Zivkovic et al. (2013), who presented values between 3.3 and 3.7 g/100 g flesh. A similar variability (1.2–4.5 g/100 g flesh) can be found in related—but better nutritionally mapped-crucian carp (*Carassius carassius*) (Lazos, Aggelousis & Alexakis, 1989; Aggelousis & Lazos, 1991; Donmez, 2009). The fat content and FA composition found in the present study is comparable with Prussian carp analyzed by Ozparlak (2013) with a low lipid content and a relatively high proportion of n-3 PUFA. Significant changes in lipid content, ash and moisture were noted, whereas no changes were seen in protein and dry matter. In some FAs including 14:0 (*p* = 0.03, *R*_2_ = 0.35); 18:3n-3 (*p* = 0.03, *R*_2_ = 0.36); 22:1 (*p* = 0.02, *R*_2_ =0.43) a positive correlation to the fat content was observed, while in the 18:1n-9 (*p* = 0.02, *R*_2_ = 0.39); 20:4n-6 (*p* = 0.01, *R*_2_ = 0.46); 20:5n-3 (*p* = 0.01, *R*_2_ = 0.48); 22:6n-3 (*p* = 0.006, *R*_2_ = 0.53) a negative correlation was observed. Kaya & Erdem (2009) observed comparable values to our findings for a fillet fat content in wild brown trout throughout the year (1.85 ± 0.1 g/100 g of flesh in January to 3.57 ± 0.2 g/100 g in June). Very similar results were also published by Kaushik et al. (2006), confirming that 2.5–3.5 g/100 g flesh is most likely a normal average fat content of wild brown trout across Europe. Also the n-3/n-6 ratio, which is 2.95 ± 1.39 in this study (Table 3), was similar to the values found by Kaya & Erdem (2009) in the same season (spring) showing a ratio 3–4. Meanwhile Kaushik et al. (2006) found lower values of 1.5–2. Again, this indicates a different composition of feed and confirms a generally high variability in the fillet FA composition within the same fish species. Significant changes in the ash content were observed as a result of changes in the localities. Brown trout with specific intestinal characteristics, including about 45 pyloric caeca, can digest the feed enzymatically through proteolytic activity (Burnstock, 1959) which facilitates in the absorption of the digested feed. However, most of the carnivorous species have shorter and thicker intestines compared to the herbivorous species (Smith, 1980; Rust, 2002), as well as increased enzymatic activity of for example, proteases and peptidases, which facilitates the absorption of the peptides and amino acids for the carnivorous species like common bream and rainbow trout (Rodrigues et al., 2017).

A positive correlation of FAs to fat content including 14:0 (*p* = 0.07, *R*_2_ = 0.28); 16:1 (*p* = 0.05, *R*_2_ = 0.33) was observed, whereas in 22:6n-3 (*p* = 0.09, *R*_2_ = 0.33) a negative correlation was observed.

Another aspect is the FA content in absolute amounts. Although some leaner fish (here European perch) may contain a high percentage of EPA and DHA compared to fatter species, the absolute amounts logically increase with an increasing fat content. The European Food Safety Authority (EFSA) recommends a minimum daily intake of EPA + DHA of 250 mg for normal population (EFSA, 2009). This means, considering 150 g fish as an average portion, all fish fulfil more than the minimal recommended intake of EPA and DHA and hence they can contribute to a much healthier diet. 300 g of nase would even fulfil the intake for a whole week. However, since fish consumption is low in Central Europe, it needs to be promoted and increased.

The lipid health indices are described in detail by Ulbricht & Southgate (1991), who stated that the values of IA and IT in food are good indicators for the risk of atherogenic and thromobogenic effects of foods, and subsequently the risk for the development of cardiovascular diseases (CVDs). The higher those values are, the higher the atherogenicity and thrombogenicity of the food items respectively is. Ulbricht & Southgate (1991) also provide IA and IT values for pork, beef and chicken (0.6, 0.7, and 0.5, respectively for IA and 1.4, 1.3, and 0.95 for IT, respectively). All found values of IA and IT are very close to the values stated for the so-called Eskimo diet, which is related to very low incidences of the coronary heart disease (IA 0.39 and IT 0.28) (Ulbricht & Southgate, 1991). In 1970s, Bang and Dyerberg, have investigated the low risk of CVD in the Greenland Eskimos population. They found reduced risks of CVD in connection with the consumption of high amount of fish and marine mammals in the Eskimos diet. At that time, n-3 PUFA consumption of the Eskimos was five times higher than Danish people intake. Subsequently, the so-called Eskimo diet has very low incidences of the coronary heart disease (IA 0.39 and IT 0.28) (Ulbricht & Southgate, 1991).

Moreover, the values of these two indices in our study are in agreement with the results of Rodrigues et al. (2017) and Monterio et al. (2017). Our results confirm that wild fish is clearly favorable for human nutrition.

## Conclusions

In this study the proximate and FA composition of seven wild freshwater fish species from the Czech Republic was analyzed. According to our findings we conclude that the chosen species have a standard protein content, minimum carbohydrates and relatively low contents of fat, which can, however, vary to some degree in various localities, most probably related to the availability and composition of feed. In addition, factors such as fish physiology and feeding habits as well as ecological factors including water body trophic status, could have an influence on the variation of nutritional composition of the different species.

As expected, we showed that there can be some variation of FA composition in the same species, depending on natural habitat and availability of feed. Simultaneously, we observed a very favorable FA composition with high proportions of n-3 PUFA, including EPA and DHA in all analyzed species. Consequently, the values of both IA and IT were low and close to the values of the so-called Eskimo diet.

The obtained data increased the nutritional information about the chosen species for experts as well as consumers. It would be beneficial to provide the local fishermen and anglers association with this information to promote consumption of fish in general, especially of yet underutilized species. Regarding the effects of the fat content, in some MUFA, PUFA, and SFAs there were correlations with the lipid content. The dynamic interaction between them needs more investigation, which then could partly explain the differences among the localities.

## Supplemental Information

10.7717/peerj.5729/supp-1 Supplemental Information 1 P-value and r-square from the correlation between different fatty acids and fat content of each species.

10.7717/peerj.5729/supp-2 Supplemental Information 2 Correlation between fatty acids and fat content of different species.

10.7717/peerj.5729/supp-3 Supplemental Information 3 P-value of calculated parameters for Tables 2 and 3, and Figure 1.

10.7717/peerj.5729/supp-4 Supplemental Information 4 Raw data and all calculations regarding proximate and fatty acid composition of different species.

## Additional Information and Declarations

### Data Availability

The following information was supplied regarding data availability: The raw data are provided in the Supplemental Files.
