# Supplementary material for: Nutritional value of several commercially important river fish species from the Czech Republic
Source: PeerJ. 2018 Oct 12;6:e5729. doi: 10.7717/peerj.5729 (PMC6187990; doi:10.7717/peerj.5729)
Supplement: Supplemental Information 1 [file peerj-06-5729-s001.pdf]

Abramis brama

14:0	14:1	16:1	18:1n-7	22:6n-3
p = 0.039287<br>R2= 0.43077468<br>df = 1.8<br>F = 6.054189	p = 0.022415<br>R2=0.49891262<br>df = 1,8<br>F = 7.965279	p = 0.022649<br>R2=.49771714<br>df = 1.8<br>F = 7.927280	p = 0.009457<br>R2= 0.59001625<br>df = 1.8<br>F = 11.51297	p = 0.038128<br>R2 0.43458996<br>F = 6.149024

Perch

14:0<br>p = ,099902<br>R2= ,20941353<br>df = 1,12	14:01<br>p = ,917370<br>R2=,00093464<br>df = 1,12<br>F = ,0112261	16:0<br>p = ,891806<br>R2=,00160600<br>df = 1,12<br>F = ,0193030	16:1<br>p = ,000697<br>R2= ,63039464<br>F = 20,46706	18:0<br>p = ,440749<br>R2=,05031298<br>F = ,6357419	18:1n-9<br>p =,991368<br>R2=,00001017<br>F = ,0001220
18:2n-6<br>p = ,042900<br>R2= ,29929865<br>F = 5,125699	18:3 n-3<br>p = ,898497<br>R2= ,00141259<br>F = ,0169751	20:1 n-9<br>p = ,090179<br>R2=,22058278<br>F = 3,396119	20:2 n-6<br>p = ,176663<br>R2= ,14657433<br>F = 2,060979	20:4n-6<br>p = ,985878<br>R2= ,00002722<br>F = ,0003266	20:3 n-3<br>p = ,402571<br>R2 ,05903940<br>F = ,7529250
20:5n-3<br>p = ,594769<br>R2= ,02428011<br>F = ,2986116	24:1<br>p = ,208759<br>R2= ,12820015<br>F = 1,764627	22:5 n-3<br>p = ,272574<br>R2 ,09925736<br>F = 1,322340	22:6n-3<br>p = ,410547<br>R2= ,05712410<br>F = ,7270196		

Carass

14:0<br>p = ,039435<br>R2= ,35921244<br>F = 5,605796	14:1<br>p = ,421871<br>R2= ,06554481<br>F = ,7014227	16:0<br>p = ,311288<br>R2=,10211872<br>F = 1,137330	16:1<br>p = ,201480<br>R2= ,15750257<br>F = 1,869473	18:0<br>p = ,071973<br>R2= ,28809657<br>F = 4,046849	18:1 n-9<br>p = ,029884<br>R2 ,39022885<br>F = 6,399595
18:1 n-3<br>p = ,020504<br>R2= ,61913692<br>F = 9,753693	18:2 n-6<br>p = ,993256<br>R2= ,00000751<br>F = ,0000751	18:3 n-3<br>p = ,037589<br>R2= ,36465674<br>F = 5,739523	20:1 n-9<br>p = ,632016<br>R2= ,02381684<br>F = ,2439792	20:2 n-6<br>p = ,858091<br>R2= ,00335507<br>F = ,0336637	20:4 n-6<br>p = ,015052<br>R2= ,46185692<br>F = 8,582418
20:3 n-3<br>p = ,189135<br>R2= ,16566292<br>F = 1,985563	22:1<br>p = ,020159<br>R2: ,43223315<br>F = 7,612864	20:5 n-3<br>p = ,011494<br>R2= ,48802685<br>F = 9,532274	24:1<br>p = ,363298<br>R2= ,08317934<br>F = ,9072586	22:5 n-3<br>p = ,872175<br>R2= ,00271752<br>F = ,0272493	22:6 n-3<br>p = ,006590<br>R2= ,53853314<br>F = 11,67003

Condrostoma

14:0<br>p = ,742552<br>R2= ,01259117<br>F = ,1147656	14:1<br>p = ,664656<br>R2= ,02182595<br>F = ,2008165	16:0<br>p = ,902991<br>R2: ,00174320<br>F = ,0157162	16:1<br>p = ,291757<br>R2= ,12230605<br>F = 1,254144	18:0<br>p = ,027921<br>R2=,43224016<br>F = 6,851773	18:1n-9<br>p = ,607917<br>R2=,03043645<br>F = ,2825272
18:1 n-3<br>p = ,234282<br>R2= ,26773939<br>F = 1,828170	18: 2n-6<br>p = ,779006<br>R2= ,00920513<br>F = ,0836158	20:1 n-9<br>p = ,239233<br>R2= ,16813878<br>F = 1,616989	20:2 n-6<br>p = ,404969<br>R2= ,07819224<br>F = ,7634240	20:4 n-6<br>p = ,003143<br>R2 ,68438960<br>F = 17,3477	20:3 n-3<br>p = ,150595<br>R2= ,21524384<br>F = 2,468530
20:5 n-3<br>p = ,432936<br>R2= ,10529582<br>F = ,7061271	22:5 n-3<br>p = ,013337<br>R2= ,51166145<br>F = 9,429837	22:6 n-3<br>p = ,010248<br>R2= ,5375686<br>F = 10,46235			

Brown trout

14:0<br>p = ,074021<br>R2= ,28466170<br>F = 3,979400<br>df = 1,10	14:1<br>p = ,393908<br>R2= ,07353521<br>F = ,7937184	16:0<br>p = ,529077<br>R2= ,04545170<br>F = ,4285434	16:1<br>p = ,050501<br>R2= ,33058520<br>F = 4,93842	18:0<br>p = ,530082<br>R2= ,04058717<br>F = ,4230418
18:1 n-9<br>p = ,253298<br>R2= ,15924535<br>F = 1,515261	18:1 n-3<br>p = ,202582<br>R2= ,25426052<br>F = 2,045705	18:2 n-6<br>p = ,308079<br>R2= ,10340862<br>F = 1,153353	18: 3n-3<br>p = ,536171<br>R2= ,03942190<br>F = ,4103977	20:1 n-9<br>p = ,189204<br>R2= ,16561554<br>F = 1,984883
20:2 n-6<br>p = ,380144<br>R2= ,07774860<br>F = ,8430305	20:4 n-6<br>p = ,150853<br>R2= ,19484356<br>F = 2,419946	20:3 n-3<br>p = ,151226<br>R2= ,19452575<br>F = 2,415046	20: 5 n-3<br>p = ,854535<br>R2= ,00352693<br>F = ,0353941	24:1<br>p = ,278746<br>R2= ,11596463<br>F = 1,311765
22:5 n-3<br>p = ,626837<br>R2= ,02453860<br>F = ,2515589	22:6 n-3<br>p = ,099222<br>R2= ,24825570<br>F = 3,302396			

Grayling

14:0<br>p = ,468923<br>R2= ,04453711<br>df = 1,12<br>F = ,5593575	16:0<br>p = ,059814<br>R2= ,26465506<br>F = 4,318872	16:1<br>p = ,067635<br>R2 ,25159889<br>F = 4,034183	18:0<br>p = ,014046<br>R2= ,40730594<br>F = 8,246533	18:1 n-9<br>p = ,004637<br>R2= ,50074156<br>F = 12,03565	18: 2 n-6<br>p = ,065092<br>R2= ,25568345<br>F = 4,122173
18: 3 n-3<br>p = ,006649<br>R2= ,47186529<br>F = 10,72148	20:1 n-9<br>p = ,233823<br>R2= ,1158128<br>F = 1,571787	20: 2n-6<br>p = ,758622<br>R2= ,00816956<br>F = ,0988422	20: 4 n-6<br>p = ,001826<br>R2= ,56897767<br>F = 15,84079	20: 3 n-3<br>p = ,594588<br>R2= ,0243036<br>F = ,2989080	20: 5 n-3<br>p = ,002458<br>R2= ,54819675<br>F = 14,56023
22:5 n-3<br>p = ,000613<br>R2= ,63795712<br>F = 21,14524	22:6 n-3<br>p = ,000806<br>R2= ,62164939<br>F = 19,71661				

Chub

14:0<br><br>p = ,002848<br><br>R2= ,69177811<br><br>F = 17,95533<br><br>df = 1,8	14:1<br><br>p = ,000571<br><br>R2= ,79111639<br><br>F = 30,29884	16:0<br><br>p = ,002482<br><br>R2= ,70177548<br><br>F = 18,82543	16:1<br><br>p = ,364614<br><br>R2= ,10352558<br><br>F = ,9238463
18:0<br><br>p = ,383722<br><br>R2= ,09596551<br><br>F = ,8492199	18: 1n-9<br><br>p = ,020749<br><br>R2= ,5077309<br><br>F = 8,251274	18: 2 n-6<br><br>p = ,029839<br><br>R2= ,46507065<br><br>F = 6,955246	18: 3 n-3<br><br>p = ,010609<br><br>R2= ,5788042<br><br>F = 10,99354
20: 1n-9<br><br>p = ,005950<br><br>R2= ,63251391<br><br>F = 13,76953	20:2 n-6<br><br>p = ,336940<br><br>R2= ,11537026<br><br>F = 1,043332	20: 4 n-6<br><br>p = ,100015<br><br>R2= ,30177142<br><br>F = 3,457566	20: 3 n-3<br><br>p = ,077172<br><br>R2= ,33939969<br><br>F = 4,110197
20: 5 n-3<br><br>p = ,095855<br><br>R2= ,30802360<br><br>F = 3,561088	24:1<br><br>p = ,003552<br><br>R2: ,67500991<br><br>F = 16,61613	22: 5 n-3<br><br>p = ,073519<br><br>R2= ,34629078<br><br>F = 4,237857	22: 6 n-3<br><br>p = ,000095<br><br>R2= ,86542638<br><br>F = 51,44701
