# Supplementary material for: Nutritional value of several commercially important river fish species from the Czech Republic
Source: PeerJ. 2018 Oct 12;6:e5729. doi: 10.7717/peerj.5729 (PMC6187990; doi:10.7717/peerj.5729)
Supplement: Supplemental Information 3 [file peerj-06-5729-s003.pdf]

```
FA_14_1

data:  FA by Species
Kruskal-Wallis chi-squared = 23.334, df = 6, p-value =
0.0006921


FA_16_0

data:  FA by Species
Kruskal-Wallis chi-squared = 42.942, df = 6, p-value =
1.198e-07

FA_16_1

data:  FA by Species
Kruskal-Wallis chi-squared = 60.047, df = 6, p-value =
4.404e-11


FA_18_0

Kruskal-Wallis rank sum test

data:  FA by Species
Kruskal-Wallis chi-squared = 28.698, df = 6, p-value =
6.938e-05


FA_18_1n_9

data:  FA by Species
Kruskal-Wallis chi-squared = 59.051, df = 6, p-value =
7.015e-11


FA_18_1n_7

data:  FA by Species
Kruskal-Wallis chi-squared = 32.905, df = 6, p-value =
1.094e-05


FA_18_2n_6

data:  FA by Species
Kruskal-Wallis chi-squared = 60.422, df = 6, p-value =
3.695e-11


FA_18_3n_3

data:  FA by Species
Kruskal-Wallis chi-squared = 38.726, df = 6, p-value =
8.098e-07
```

FA_20

data: FA by Species
Kruskal-Wallis chi-squared = 22.911, df = 6, p-value =
0.0008267

FA 20_1n_9

data: FA by Species
Kruskal-Wallis chi-squared = 62.335, df = 6, p-value =
1.508e-11

FA_20_2n_6

data: FA by Species
Kruskal-Wallis chi-squared = 47.176, df = 6, p-value =
1.726e-08

FA_20_4n_6

data: FA by Species
Kruskal-Wallis chi-squared = 59.622, df = 6, p-value =
5.371e-11

FA_20_3n_3

data: FA by Species
Kruskal-Wallis chi-squared = 58.423, df = 6, p-value =
9.404e-11

FA_22

data: FA by Species
Kruskal-Wallis chi-squared = 22.911, df = 6, p-value =
0.0008267

FA_22_1
data: FA by Species
Kruskal-Wallis chi-squared = 20.275, df = 6, p-value =
0.002474

FA 20_5n_3
data: FA by Species
Kruskal-Wallis chi-squared = 26.107, df = 6, p-value =
0.0002126

FA 24_1
data: FA by Species
Kruskal-Wallis chi-squared = 8.8036, df = 6, p-value =
0.1849

FA_22_5n_3
data: FA by Species

Kruskal-Wallis chi-squared = 56.728, df = 6, p-value = 2.074e-10

FA 22_6n_3
data: FA by Species
Kruskal-Wallis chi-squared = 46.083, df = 6, p-value = 2.85e-08

FA 24_0
data: FA by Species
Kruskal-Wallis chi-squared = 39.077, df = 6, p-value = 6.912e-07

SFA
data: FA by Species
Kruskal-Wallis chi-squared = 34.684, df = 6, p-value = 4.962e-06

MUFA
data: FA by Species
Kruskal-Wallis chi-squared = 38.013, df = 6, p-value = 1.117e-06

PUFA

data: FA by Species
Kruskal-Wallis chi-squared = 40.578, df = 6, p-value = 3.506e-07

N_3_PUFA

data: FA by Species
Kruskal-Wallis chi-squared = 44.837, df = 6, p-value = 5.042e-08

N_6_PUFA

data: FA by Species
Kruskal-Wallis chi-squared = 52.45, df = 6, p-value = 1.514e-09

N_3_HUFA

Kruskal-Wallis rank sum test

data: FA by Species
Kruskal-Wallis chi-squared = 44.837, df = 6, p-value = 5.042e-08

N_3_N_6

data: FA by Species
Kruskal-Wallis chi-squared = 56.829, df = 6, p-value = 1.979e-10

IA
data: FA by Species
Kruskal-Wallis chi-squared = 29.489, df = 6, p-value =
4.915e-0
IT
data: FA by Species
Kruskal-Wallis chi-squared = 19.935, df = 6, p-value =
0.002844

Dry matter

data: Dry by Species
Kruskal-Wallis chi-squared = 40.786, df = 6, p-value = 3.19e-07

Lipids

data: Lipids by Species
Kruskal-Wallis chi-squared = 45.88, df = 6, p-value = 3.128e-08

data: energy by Species

Kruskal-Wallis chi-squared = 23.579, df = 6, p-value = 0.0006241

>
Kruskal-Wallis rank sum test

data: EPA+DHA by Species
Kruskal-Wallis chi-squared = 34.14, df = 6, p-value = 6.32e-06

Kruskal-Wallis rank sum test
